# Supplementary material for: Linguistic structure and language familiarity sharpen phoneme encoding in the brain
Source: Commun Biol. 2026 Mar 20;9:638. doi: 10.1038/s42003-026-09865-8 (PMC13168568; doi:10.1038/s42003-026-09865-8)
Supplement: Supplementary file 1 — Supplemental Material [file 42003_2026_9865_MOESM1_ESM.pdf]

SUPPLEMENTARY INFORMATION

Supplementary Table 1. Accuracy improvement by each feature in sentences and words in Dutch stimuli

Features	t-statistic	df	p-value
Sentences Acoustic Edge	5.87	19	< 0.0001
Sentences Phoneme Features	6.87	19	< 0.0001
Sentences Word Features	6.06	19	< 0.0001
Words Acoustic Edge	6.79	19	< 0.0001
Words Phoneme Features	6.47	19	< 0.0001
Words Word Features	5.00	19	< 0.0001

Supplementary Table 2. Accuracy improvement by each feature in sentences and words in Turkish stimuli

Features	t-statistic	df	p-value
Sentences Acoustic Edge	6.72	29	< 0.0001
Sentences Phoneme Features	10.87	29	< 0.0001
Sentences Word Features	9.39	29	< 0.0001
Words Acoustic Edge	7.42	29	< 0.0001
Words Phoneme Features	10.73	29	< 0.0001
Words Word Features	10.74	29	< 0.0001

Supplementary Table 3. LME results of sentences vs words in Dutch stimuli

	Sum Sq	Mean Sq	NumDF	DenDF	F value	Pr(>F)
Condition	3.60E-08	3.60E-08	1	55	0.0874	0.7686
Feature	3.94E-05	3.94E-05	1	55	94.6701	1.48E-13

contrast	estimate	SE	df	t.ratio	p.value
Sentences Acoustic Edge - Words Acoustic Edge	4.38E-05	1.48E-04	55.00	0.296	0.99090
Sentences Acoustic Edge - Sentences Phoneme Onset	1.44E-03	1.48E-04	55.00	9.730	<.0001
Sentences Acoustic Edge - Words Phoneme Onset	1.48E-03	2.09E-04	55.00	7.089	<.0001
Words Acoustic Edge - Sentences Phoneme Onset	1.40E-03	2.09E-04	55.00	6.671	<.0001
Words Acoustic Edge - Words Phoneme Onset	1.44E-03	1.48E-04	55.00	9.730	<.0001
Sentences Phoneme Onset - Words Phoneme Onset	4.38E-05	1.48E-04	55.00	0.296	0.99090

Supplementary Table 4. LME results of sentences vs words for Turkish stimuli

	Sum Sq	Mean Sq	NumDF	DenDF	F value	Pr(>F)
Condition	2.20E-07	2.20E-07	1	59.00	6.68	1.22E-02
Feature	5.84E-08	5.84E-08	1	29.00	1.78	1.93E-01

contrast	estimate	SE	df	t.ratio	p.value
Sentences Acoustic Edge - Words Acoustic Edge	8.56E-05	3.31E-05	59.00	2.585	0.05760
Sentences Acoustic Edge - Sentences Phoneme Onset	7.96E-05	5.98E-05	29.00	1.333	0.55020
Sentences Acoustic Edge - Words Phoneme Onset	1.65E-04	6.83E-05	47.40	2.418	0.08730
Words Acoustic Edge - Sentences Phoneme Onset	-5.93E-06	6.83E-05	47.40	-0.087	0.99980
Words Acoustic Edge - Words Phoneme Onset	7.96E-05	5.98E-05	29.00	1.333	0.55020
Sentences Phoneme Onset - Words Phoneme Onset	8.56E-05	3.31E-05	59.00	2.585	0.05760

Supplementary Table 5. Accuracy improvement by each feature in words and syllables for Dutch-speaking participants

Features	t-statistic	df	p-value
Dutch Stimuli Words Acoustic Edge	6.63	14	< 0.0001
Dutch Stimuli Words Phoneme Features	3.45	14	0.00393
Chinese Stimuli Words Acoustic Edge	7.02	13	< 0.0001
Chinese Stimuli Words Phoneme Features	7.48	13	< 0.0001
Dutch Stimuli Syllables Acoustic Edge	5.93	14	< 0.0001
Dutch Stimuli Syllables Phoneme Features	4.02	14	0.00126
Chinese Stimuli Syllables Acoustic Edge	6.25	13	< 0.0001
Chinese Stimuli Syllables Phoneme Features	5.37	13	< 0.0001

Supplementary Table 6. Accuracy improvement by each feature in words and syllables for Mandarin Chinese-speaking participants

Features	t-statistic	df	p-value
Dutch Stimuli Words Acoustic Edge	4.58	14	0.00052
Dutch Stimuli Words Phoneme Features	7.37	14	< 0.0001
Chinese Stimuli Words Acoustic Edge	4.64	13	0.00046
Chinese Stimuli Words Phoneme Features	5.06	13	0.00021
Dutch Stimuli Syllables Acoustic Edge	4.19	14	0.00107
Dutch Stimuli Syllables Phoneme Features	4.03	14	0.00143
Chinese Stimuli Syllables Acoustic Edge	4.56	13	0.00011
Chinese Stimuli Syllables Phoneme Features	4.41	13	0.00071

Supplementary Table 7. Accuracy improvement by each feature in words and syllables for Turkish-speaking participants

Features	t-statistic	df	p-value
Words Acoustic Edge	7.56	29	< 0.0001
Words Phoneme Features	9.15	29	< 0.0001
Syllables Acoustic Edge	6.52	29	< 0.0001
Syllables Phoneme Features	8.87	29	< 0.0001

Supplementary Table 8. LME results of words vs syllables for Mandarin-speaking participants – Mandarin Chinese stimuli

	Sum Sq	Mean Sq	NumDF	DenDF	F value	Pr(>F)
Condition	1.15E-05	1.15E-05	1	26.00	13.37	1.14E-03
Feature	2.73E-06	2.73E-06	1	13.00	3.17	9.86E-02
Condition:Feature	1.74E-05	1.74E-05	1	26.00	20.16	1.29E-04

contrast	estimate	SE	df	t.ratio	p.value
Syllables Acoustic Edge - Words Acoustic Edge	2.07E-04	3.51E-04	26	0.59	0.93440
Syllables Acoustic Edge -Syllables Phoneme Features	1.91E-03	5.13E-04	21.1	3.73	0.00620
Syllables Acoustic Edge - Words Phoneme Features	-1.08E-04	5.13E-04	21.1	-0.21	0.99660
Words Acoustic Edge -Syllables Phoneme Features	1.71E-03	5.13E-04	21.1	3.33	0.01560
Words Acoustic Edge - Words Phoneme Features	-3.14E-04	5.13E-04	21.1	-0.61	0.92690
Syllables Phoneme Features- Words Phoneme Features	-2.02E-03	3.51E-04	26	-5.76	<.0001

Supplementary Table 9. LME results of words vs syllables for Mandarin-speaking participants – Dutch stimuli

	Sum Sq	Mean Sq	NumDF	DenDF	F value	Pr(>F)
Condition	1.77E-05	1.77E-05	1	26.00	44.61	4.38E-07
Feature	7.40E-07	7.40E-07	1	13.00	1.87	1.95E-01
Condition:Feature	1.58E-05	1.58E-05	1	26.00	39.75	1.13E-06

contrast	estimate	SE	df	t.ratio	p.value
Syllables Acoustic Edge - Words Acoustic Edge	-6.30E-05	2.38E-04	26	-0.26	0.99340
Syllables Acoustic Edge -Syllables Phoneme Features	1.47E-03	3.40E-04	21.7	4.31	0.00160
Syllables Acoustic Edge - Words Phoneme Features	-7.21E-04	3.40E-04	21.7	-2.12	0.17900
Words Acoustic Edge -Syllables Phoneme Features	1.53E-03	3.40E-04	21.7	4.49	0.00100
Words Acoustic Edge - Words Phoneme Features	-6.58E-04	3.40E-04	21.7	-1.93	0.24410

Syllables Phoneme Features- Words Phoneme Features	-2.19E-03	2.38E-04	26	-9.18	<.0001

Supplementary Table 10. LME results of words vs syllables for Dutch-speaking participants – Mandarin Chinese stimuli

	**Sum Sq**	**Mean Sq**	**NumDF**	**DenDF**	**F value**	**Pr(>F)**
Condition	1.26E-06	1.26E-06	1	29.00	19.80	1.17E-04
Feature	1.58E-06	1.58E-06	1	14.00	24.87	1.99E-04

**contrast**	**estimate**	**SE**	**df**	**t.ratio**	**p.value**
Syllables Acoustic Edge - Words Acoustic Edge	-2.26E-04	9.19E-05	28	-2.46	0.08850
Syllables Acoustic Edge -Syllables Phoneme Features	7.97E-04	1.61E-04	19.2	4.96	0.00050
Syllables Acoustic Edge - Words Phoneme Features	4.44E-04	1.55E-04	14	2.86	0.05460
Words Acoustic Edge -Syllables Phoneme Features	1.02E-03	1.67E-04	14	6.14	0.00010
Words Acoustic Edge - Words Phoneme Features	6.70E-04	1.61E-04	19.2	4.17	0.00260
Syllables Phoneme Features- Words Phoneme Features	-3.53E-04	9.19E-05	28	-3.84	0.00340

Supplementary Table 11. LME results of words vs syllables for Dutch-speaking participants – Dutch stimuli

	**Sum Sq**	**Mean Sq**	**NumDF**	**DenDF**	**F value**	**Pr(>F)**
Condition	2.51E-05	2.51E-05	1	42.00	13.32	7.21E-04
Feature	1.47E-06	1.47E-06	1	42.00	0.78	3.82E-01
Condition:Feature	2.10E-05	2.10E-05	1	42.00	11.14	1.78E-03

**contrast**	**estimate**	**SE**	**df**	**t.ratio**	**p.value**
Syllables Acoustic Edge - Words Acoustic Edge	-1.10E-04	5.01E-04	42	-0.22	0.99620
Syllables Acoustic Edge -Syllables Phoneme Features	8.70E-04	5.01E-04	42	1.74	0.31870
Syllables Acoustic Edge - Words Phoneme Features	-1.61E-03	5.01E-04	42	-3.21	0.01320
Words Acoustic Edge -Syllables Phoneme Features	9.80E-04	5.01E-04	42	1.96	0.22110
Words Acoustic Edge - Words Phoneme Features	-1.50E-03	5.01E-04	42	-2.99	0.02340
Syllables Phoneme Features- Words Phoneme Features	-2.48E-03	5.01E-04	42	-4.94	0.00010

Supplementary Table 12. LME results of words vs syllables for Turkish-speaking participants – Turkish stimuli

	**Sum Sq**	**Mean Sq**	**NumDF**	**DenDF**	**F value**	**Pr(>F)**
Condition	1.26E-04	1.26E-04	1	87.00	110.618	< 2.2e-16
Feature	7.64E-05	7.64E-05	1	30.00	67.228	3.76E-09

Condition:Feature	1.12E-04	1.12E-04	1	87.00	98.418	5.87E-16

contrast	estimate	SE	df	t.ratio	p.value
Syllables Acoustic Edge - Words Acoustic Edge	-1.16E-04	2.75E-04	58.00	-0.422	0.97450
Syllables Acoustic Edge -Syllables Phoneme Features	-1.40E-03	4.50E-04	42.70	-3.104	0.01710
Syllables Acoustic Edge - Words Phoneme Features	-5.38E-03	4.50E-04	42.70	-11.941	<.0001
Words Acoustic Edge -Syllables Phoneme Features	-1.28E-03	4.50E-04	42.70	-2.846	0.03300
Words Acoustic Edge - Words Phoneme Features	-5.26E-03	4.50E-04	42.70	-11.683	<.0001
Syllables Phoneme Features- Words Phoneme Features	-3.98E-03	2.75E-04	58.00	-14.452	<.0001

Supplementary Table 13. LME results of words condition for nonnative language familiarity accuracy comparison

	Sum Sq	Mean Sq	NumDF	DenDF	F value	Pr(>F)
Stimuli	6.94E-06	6.94E-06	1	81.00	3.83	5.38E-02
Feature	5.85E-06	5.85E-06	1	81.00	3.23	7.60E-02
Group	6.43E-06	6.43E-06	1	27.00	3.55	7.04E-02
Stimuli:Feature	6.02E-06	6.02E-06	1	81.00	3.32	7.20E-02
Stimuli:Group	4.72E-06	4.72E-06	1	81.00	2.60	1.10E-01
Feature:Group	3.86E-08	3.86E-08	1	81.00	0.02	8.84E-01
Stimuli:Feature:Group	1.14E-05	1.14E-05	1	81.00	6.30	1.41E-02

contrast	estimate	SE	df	t.ratio	p.value
Native Acoustic Edge Chinese_group - Nonnative Acoustic Edge Chinese_group	2.57E-04	5.09E-04	81	0.51	0.9996
Native Acoustic Edge Chinese_group - Native Phoneme Features Chinese_group	-3.14E-04	5.09E-04	81	-0.62	0.9985
Native Acoustic Edge Chinese_group - Nonnative Phoneme Features Chinese_group	-4.00E-04	5.09E-04	81	-0.79	0.9934
Native Acoustic Edge Chinese_group - Native Acoustic Edge Dutch_group	8.82E-04	5.69E-04	93.6	1.55	0.7780
Native Acoustic Edge Chinese_group - Nonnative Acoustic Edge Dutch_group	6.91E-04	5.69E-04	93.6	1.22	0.9254
Native Acoustic Edge Chinese_group - Native Phoneme Features Dutch_group	-6.15E-04	5.69E-04	93.6	-1.08	0.9593
Native Acoustic Edge Chinese_group - Nonnative Phoneme Features Dutch_group	1.36E-03	5.69E-04	93.6	2.39	0.2565
Nonnative Acoustic Edge Chinese_group - Native Phoneme Features Chinese_group	-5.72E-04	5.09E-04	81	-1.12	0.9497

Nonnative Acoustic Edge Chinese_group - Nonnative Phoneme Features Chinese_group	-6.58E-04	5.09E-04	81	-1.29	0.8990
Nonnative Acoustic Edge Chinese_group - Native Acoustic Edge Dutch_group	6.24E-04	5.69E-04	93.6	1.10	0.9558
Nonnative Acoustic Edge Chinese_group - Nonnative Acoustic Edge Dutch_group	4.34E-04	5.69E-04	93.6	0.76	0.9946
Nonnative Acoustic Edge Chinese_group - Native Phoneme Features Dutch_group	-8.72E-04	5.69E-04	93.6	-1.53	0.7875
Nonnative Acoustic Edge Chinese_group - Nonnative Phoneme Features Dutch_group	1.10E-03	5.69E-04	93.6	1.94	0.5265
Native Phoneme Features Chinese_group - Nonnative Phoneme Features Chinese_group	-8.58E-05	5.09E-04	81	-0.17	1.0000
Native Phoneme Features Chinese_group - Native Acoustic Edge Dutch_group	1.20E-03	5.69E-04	93.6	2.10	0.4207
Native Phoneme Features Chinese_group - Nonnative Acoustic Edge Dutch_group	1.01E-03	5.69E-04	93.6	1.77	0.6428
Native Phoneme Features Chinese_group - Native Phoneme Features Dutch_group	-3.00E-04	5.69E-04	93.6	-0.53	0.9995
Native Phoneme Features Chinese_group - Nonnative Phoneme Features Dutch_group	1.68E-03	5.69E-04	93.6	2.95	0.0748
Nonnative Phoneme Features Chinese_group - Native Acoustic Edge Dutch_group	1.28E-03	5.69E-04	93.6	2.25	0.3302
Nonnative Phoneme Features Chinese_group - Nonnative Acoustic Edge Dutch_group	1.09E-03	5.69E-04	93.6	1.92	0.5416
Nonnative Phoneme Features Chinese_group - Native Phoneme Features Dutch_group	-2.14E-04	5.69E-04	93.6	-0.38	0.9999
Nonnative Phoneme Features Chinese_group - Nonnative Phoneme Features Dutch_group	1.76E-03	5.69E-04	93.6	3.10	0.0504
Native Acoustic Edge Dutch_group - Nonnative Acoustic Edge Dutch_group	-1.90E-04	4.92E-04	81	-0.39	0.9999
Native Acoustic Edge Dutch_group - Native Phoneme Features Dutch_group	-1.50E-03	4.92E-04	81	-3.05	0.0597
Native Acoustic Edge Dutch_group - Nonnative Phoneme Features Dutch_group	4.80E-04	4.92E-04	81	0.98	0.9765
Nonnative Acoustic Edge Dutch_group - Native Phoneme Features Dutch_group	-1.31E-03	4.92E-04	81	-2.66	0.1513
Nonnative Acoustic Edge Dutch_group - Nonnative Phoneme Features Dutch_group	6.70E-04	4.92E-04	81	1.36	0.8706
Native Phoneme Features Dutch_group - Nonnative Phoneme Features Dutch_group	1.98E-03	4.92E-04	81	4.02	0.0031

Supplementary Table 14. ANOVA results of words condition for nonnative language familiarity comparison of peak times

	Df	Df.res	F value	Pr(>F)
Stimuli	1	324	15.23	0.0001
Feature	1	324	5.81	0.0165
Group	1	324	3.92	0.0486
Time	2	324	1298.30	<0.0001
Stimuli:Feature	1	324	3.28	0.0713
Stimuli:Group	1	324	10.45	0.0014
Feature:Group	1	324	0.03	0.8517
Stimuli:Time	2	324	8.63	0.0002
Feature:Time	2	324	5.68	0.0038
Group:Time	2	324	3.54	0.0301
Stimuli:Feature:Group	1	324	0.51	0.4752
Stimuli:Feature:Time	2	324	3.46	0.0327
Stimuli:Group:Time	2	324	3.66	0.0268
Feature:Group:Time	2	324	0.14	0.8717
Stimuli:Feature:Group:Time	2	324	2.58	0.0775

Contrast	Group	Time	Feature	estimate	SE	df	t.ratio	p.value
Native - Nonnative	Mandarin	Early	Acoustic Edge	-52.60	37.9	324.0	-1.39	0.1663
Native - Nonnative	Dutch	Early	Acoustic Edge	-31.30	36.6	324.0	-0.86	0.3932
Native - Nonnative	Mandarin	Late	Acoustic Edge	-30.00	37.9	324.0	-0.79	0.4291
Native - Nonnative	Dutch	Late	Acoustic Edge	-36.50	36.6	324.0	-1.00	0.3191
Native - Nonnative	Mandarin	Middle	Acoustic Edge	-46.60	37.9	324.0	-1.23	0.2200
Native - Nonnative	Dutch	Middle	Acoustic Edge	-42.50	36.6	324.0	-1.16	0.2469
Native - Nonnative	Mandarin	Early	Phonemes	-35.00	37.9	324.0	-0.92	0.3564
Native - Nonnative	Dutch	Early	Phonemes	-42.00	36.6	324.0	-1.15	0.2521
Native - Nonnative	Mandarin	Late	Phonemes	-69.40	37.9	324.0	-1.83	0.0681
Native - Nonnative	Dutch	Late	Phonemes	-60.10	36.6	324.0	-1.64	0.1016

Native - Nonnative	Mandarin	Middle	Phonemes	-46.40	37.9	324.0	-1.23	0.2214
Native - Nonnative	Dutch	Middle	Phonemes	-11.40	36.6	324.0	-0.31	0.7557

Supplementary Table 15. Model names and speech features in models for Sentences vs. Word List comparison

	Spectrogram	Acoustic Edge	Phoneme Onset	Phoneme Surprisal	Phoneme Entropy	Word Surprisal	Word Entropy
W/o Acoustic	✓		✓	✓	✓	✓	✓
W/o Phoneme Onset , Surprisal and Entropy	✓	✓				✓	✓
Full model	✓	✓	✓	✓	✓	✓	✓

Supplementary Table 16. Model names and speech features in models for Words vs. Random Syllable Streams comparison

	Spectrogram	Acoustic Edge	Phoneme Onset	Phoneme Surprisal	Phoneme Entropy
W/o Acoustic	✓		✓	✓	✓
W/o Phoneme Onset , Surprisal and Entropy	✓	✓			
Full model	✓	✓	✓	✓	✓
